# Numerical optimization of process parameters in HLLW spray calcination

**DOI:** 10.1371/journal.pone.0347453

**Published:** 2026-07-29

**Authors:** Feng Gao, Yuzhou Ming, Yajun Zhang, Bochao Wang, Xingyun Jia

**Affiliations:** 1 College of Mechanical and Electrical Engineering, Beijing University of Chemical Technology, Beijing, China; 2 China Institute of Atomic Energy, Beijing, China; NED University of Engineering and Technology, PAKISTAN

## Abstract

To address the issues of incomplete reactions and potential solids accumulation in high‑level liquid waste (HLLW) spray calcination, this study developed a 3D computational domain utilizing a species transport model to simulate the combustion of a nitric acid, sucrose, and nitrate solution. The impacts of wall heating temperature (200–1200 °C), porous media porosity (0–1), inlet velocity (0.001–0.2 m/s), and reactant concentrations on the sodium oxide (Na_2_O) product mass fraction were systematically investigated. Simulation results demonstrate: (1) wall temperature positively correlates with Na_2_O formation, with the mass fraction rising from 0.089 to 0.360 as temperature increases from 200 °C to 1200 °C; (2) higher porosity facilitates product formation, though growth stagnates within the 0.2–0.8 range; (3) inlet velocity dictates reaction kinetics, peaking at an optimal 0.01 m/s (Na_2_O mass fraction 0.287), whereas velocities >0.05 m/s cause incomplete reactions; and (4) doubling sucrose concentration boosts Na_2_O yield by 115% and 180% more than equivalent increases in nitric acid and nitrates, respectively. Enhancing solid product recovery requires elevating wall temperature, regulating inlet velocity at approximately 0.01 m/s, and optimizing the sucrose reductant ratio.

## 1. Introduction

With the increasing global demand for low-carbon energy, nuclear power has played an increasingly prominent role in ensuring energy security and addressing climate change [1]. However, the disposal of High-Level Liquid Waste (HLLW) generated during nuclear power production has emerged as a critical challenge restricting the sustainable development of nuclear energy. HLLW contains a significant amount of long-lived radionuclides and highly toxic substances, the safe management of which is directly related to ecological security [2,3]. Currently, vitrification is recognized as the mainstream technology for HLLW treatment due to the excellent chemical, thermal, and mechanical stability of the resulting glass forms, which are considered the optimal solution for achieving long-term isolation of radioactive materials [4,5].

In the vitrification process chain, denitration and calcination during the pretreatment stage are key links for improving immobilization efficiency. Spray calcination converts liquid waste into micrometer-sized droplets through high-pressure atomization, which then undergo instantaneous flash evaporation and chemical decomposition in a high-temperature environment, transforming the liquid waste into stable oxide powders [6,7]. Compared with traditional fluidized bed technology, spray calcination devices offer significant advantages, including rapid response, high thermal efficiency, and superior radioactive containment [8,9]. However, the interior of the spray calciner involves extremely complex physicochemical processes, such as the coupling of gas-liquid two-phase flow, intense phase-change heat transfer, and complex reaction kinetics involving nitrates and reducing agents (e.g., sucrose) [10,11]. In practical operation, uneven local temperature distribution often leads to incomplete chemical reactions and may promote solids accumulation near the walls, which could affect the continuous operation of the device [12,13].

Due to the extreme environment of high temperature and intense radiation inside the calciner, it is difficult to accurately capture the microscopic evolution of reactions through experimental methods alone [14]. In recent years, Computational Fluid Dynamics (CFD) has become a key tool for optimizing nuclear waste treatment processes [15,16]. Currently, several studies have analyzed droplet dynamics and heat transfer characteristics during the spraying process [17,18]. However, most models remain limited to simplified physical processes and rarely consider the coupled effects of multi-component chemical reaction kinetics and porous media filtration layers on the flow field [19,20]. Particularly for special components like HLLW, systematic theoretical and quantitative research on how to regulate the generation characteristics of products (such as Na_2_O) under the integrated action of multiple factors—including wall temperature, inlet flow velocity, and component concentration—is still lacking [21–23].

In the present study, a high-fidelity 3D CFD simulation platform was constructed based on ANSYS Fluent, coupling the Arrhenius kinetic model, the species transport model, and the porous media model [24,25]. Building upon existing research regarding the thermal flow fields of calcination devices [26–28], this work systematically analyzes the nonlinear effects of wall temperature (200–1200°C), porous media porosity, inlet velocity, and reactant concentration on calcination efficiency. The results are intended to reveal the dynamic reaction mechanisms within the calciner and provide scientific evidence and data support for the process optimization and industrial application of spray calcination devices.

## 2. Methods

### 2.1. Geometric configuration and computational domain

**Fig 1:**
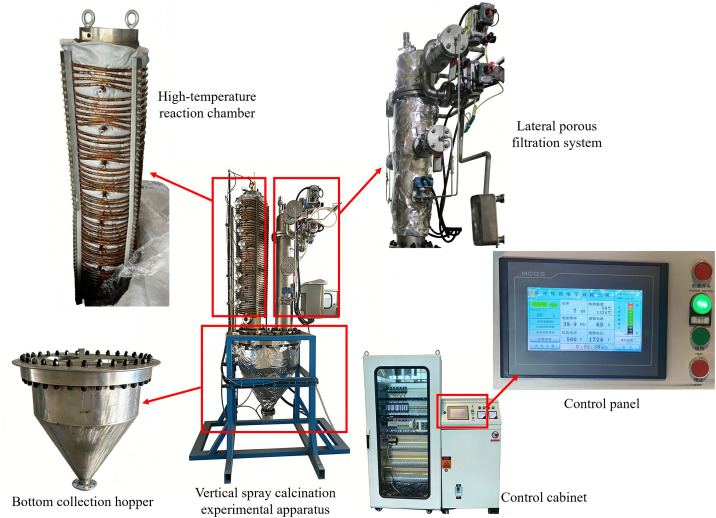
Experimental prototype of the vertical spray calciner.

The research focuses on an isometrically scaled vertical spray calcination experimental apparatus, the physical configuration of which is depicted in Fig 1.

The computational domain primarily consists of a high-temperature reaction chamber, a bottom collection hopper, and a lateral porous filtration system. To accurately characterize the physicochemical evolution of the waste liquid post-atomization, the geometric structure was reconstructed with high fidelity. The waste liquid is introduced from the top and undergoes thermal decomposition driven by intensified axial and radial thermal gradients.

**Fig 2:**
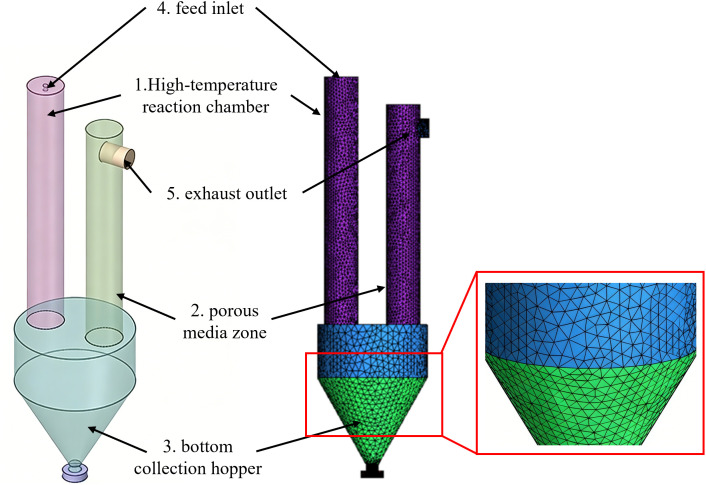
Computational mesh and discretization of the spray calcination fluid domain.

The mesh was generated using ICEM CFD with an unstructured discretization scheme. Tetrahedral elements were employed to discretize the entire fluid domain, with non-uniform sizing applied to various planes to optimize computational efficiency. Local mesh refinement was implemented at the inlet and outlet regions to capture high-gradient flow features. The mesh layout of the fluid domain is illustrated in Fig 2, where “1” represents the high-temperature reaction chamber, “3” denotes the bottom collection hopper, “4” is the feed inlet, and “5” is the exhaust outlet. The domain is categorized into two distinct regions: the porous media zone (labeled as “2”) and the general fluid zones. The final mesh comprises approximately 390,000 elements, with a minimum orthogonal quality exceeding 0.4, ensuring high grid reliability and numerical stability for the simulation.

To ensure that the numerical results are independent of the mesh discretization, a grid-convergence study was performed using five progressively refined meshes: 2.1 × 10^5^, 3.0 × 10^5^, 3.9 × 10^5^, 5.0 × 10^5^, and 7.8 × 10^5^ cells. The area-averaged Na_2_O mass fraction, which is the primary performance indicator throughout this work, was monitored under the four representative optimum conditions: wall temperature 800 °C, porous media porosity 0.8, inlet velocity 0.01 m s^-1^, and baseline reactant concentration (sucrose diluted). The results are summarized in Table 1.

**Table 1:** Grid-convergence results for Na_2_O mass fraction at different mesh sizes under representative operating conditions.

Parameter case	2.1 × 10^5^ cells	3.0 × 10^5^ cells	3.9 × 10^5^ cells	5.0 × 10^5^ cells	7.8 × 10^5^ cells
Wall temperature 800 °C	0.262	0.266	0.276	0.278	0.279
Porous media porosity 0.8	0.234	0.240	0.248	0.249	0.250
Inlet velocity 0.01 m s^-1^	0.272	0.277	0.287	0.289	0.290
Reactant concentration (sucrose, diluted)	0.288	0.294	0.304	0.306	0.307

**Fig 3:**
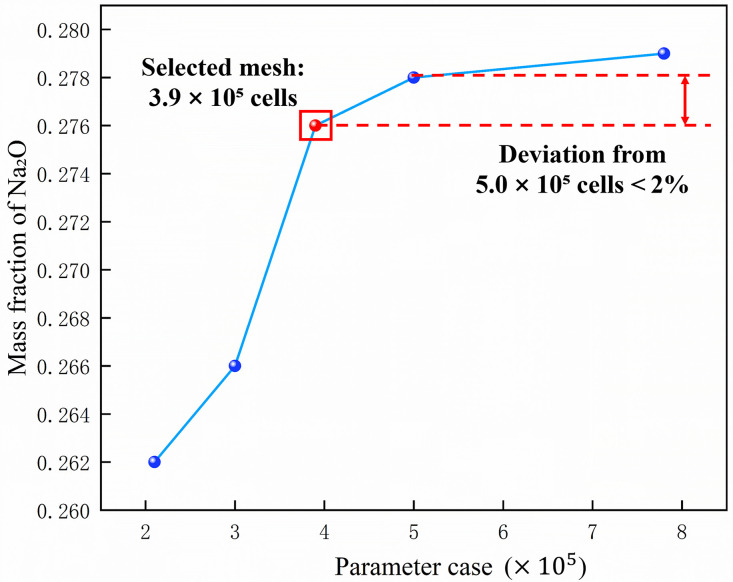
Grid-convergence curve of the area-averaged Na_2_O mass fraction (wall temperature 800 °C).

A representative convergence curve for the wall-temperature case is shown in Fig 3. As the mesh is refined beyond 3.9 × 10^5^ cells, the Na_2_O mass fraction changes by less than 2% for all cases. Even the 3.0 × 10^5^-cell mesh yields values within 5% of the reference mesh, confirming monotonic convergence. Therefore, the mesh with 3.9 × 10^5^ cells (circled in Fig 3) was adopted for all subsequent simulations, as it guarantees grid-independent results while maintaining reasonable computational cost.

It should be noted that the present steady-state model captures the fully developed thermal and flow state of the continuously operating calciner. The use of a steady-state formulation is justified by the continuous feed and heating operation, the separation of timescales between the rapid gas-phase transport (residence time ≈ 2–40 s) and the slow thermal equilibration of the apparatus (∼ 240 min), and the parametric nature of this study, which focuses on comparing equilibrium responses to different operating conditions. Transient phenomena such as start-up heating, cyclic vibration-induced solids detachment, and gradual filter fouling are not simulated in the present model and would require dedicated transient modelling in future work.

### 2.2. Governing equations and turbulence model

The spray calcination process for high-level liquid waste (HLLW) involves a complex multi-physics coupling, including droplet evaporation, gas-liquid two-phase flow, and thermochemical decomposition. Since the internal gas flow is primarily driven by a negative pressure fan—resulting in relatively low flow velocities and minimal pressure drops—the gas phase can be approximated as an incompressible Newtonian fluid. Based on the ANSYS Fluent solver, the three-dimensional steady-state Computational Fluid Dynamics (CFD) model is established as follows:

#### 2.2.1. Governing equations and turbulence model.

The gas-liquid two-phase flow and heat transfer processes within the calciner were simulated by solving the three-dimensional steady-state Reynolds-Averaged Navier-Stokes (RANS) equations. Due to its robustness in predicting fully turbulent swirling and jet flows, the Standard k−ϵ turbulence model was employed for turbulence closure, utilizing standard empirical constants ($C_{\text{1}\epsilon }$ = 1.44,$C_{\text{2}\epsilon }$ = 1.92,$C_{\mu }$ = 0.09, $\sigma_{k}$ = 1.0 and $\sigma_{\epsilon }$ = 1.3). The coupled energy conservation and species transport equations were solved to account for the spatial distribution of thermal gradients, latent heat of droplet evaporation, and multi-component chemical reactions.

All simulations were run until the scaled residuals for continuity, momentum, turbulence kinetic energy, turbulence dissipation rate, and all species transport equations fell below 10^-4^, and the residual for the energy equation fell below 10^-6^. In addition, the area-averaged Na_2_O mass fraction at the monitor point was required to remain stable with a variation of less than 0.5% over the final 500 iterations to ensure that a fully converged steady-state solution had been reached.

#### 2.2.2. Energy conservation and species transport.

To accurately characterize the internal temperature gradients and the spatial distribution of reaction products, energy conservation and species transport models are incorporated.

Energy Equation:


$$\begin{array}{c} \nabla \cdot \left(v(\rho E+p)\right)=\nabla \cdot \left(k_{eff}\nabla T-\sum_{j}h_{j}J_{j}+\left({\overset{―}{\overset{―}{\tau }}}_{eff}\cdot v\right)\right)+S_{h} \end{array}$$ (1)

Where $k_{eff}$ is the effective thermal conductivity, $J_{j}$ is the diffusion flux of species j, and $S_{h}$ accounts for the heat of chemical reactions and other volumetric heat sources.

The evolution of multiple components in the mixed waste liquid (e.g., nitric acid, sucrose, and nitrates) is determined by solving the convection-diffusion equation for the i -th species to ensure local mass conservation of its mass fraction $Y_{i}$:


$$\begin{array}{c} \nabla \cdot \left(\rho vY_{i}\right)=-\nabla \cdot J_{i}+R_{i}+S_{i} \end{array}$$ (2)

Where $R_{i}$ is the net production rate of the species determined by Arrhenius chemical reaction kinetics, and $S_{i}$ is the additional source term from discrete phase evaporation or user-defined inputs.

### 2.3. Chemical reaction kinetics model

The chemical evolution of the gas mixture during the decomposition of the nitrate system is simulated using the species transport model. The reaction rates follow the Arrhenius law, focusing on the following core thermochemical processes:


$$\begin{array}{c} \text{4}\text{8}HNO_{\text{3}}+C_{\text{1}\text{2}}H_{\text{2}\text{2}}O_{\text{1}\text{1}}=\text{4}\text{8}NO_{\text{2}}↑+\text{1}\text{2}CO_{\text{2}}↑+\text{3}\text{5}H_{\text{2}}O↑ \end{array}$$ (3)

$$\begin{array}{c} \text{4}\text{8}NaNO_{\text{3}}+C_{\text{1}\text{2}}H_{\text{2}\text{2}}O_{\text{1}\text{1}}=\text{2}\text{4}Na_{\text{2}}O+\text{4}\text{8}NO_{\text{2}}↑+\text{1}\text{2}CO_{\text{2}}↑+\text{1}\text{1}H_{\text{2}}O \end{array}$$ (4)

The two global reactions selected for this study represent the principal thermochemical pathways in the calcination of HLLW using sucrose as a denitration agent. The first reaction (HNO_3_ decomposition) describes the elimination of nitric acid, which serves both as a solvent and as a strong oxidiser in the waste stream. The second reaction (NaNO_3_ carbothermic reduction) represents the primary pathway for converting dissolved nitrate salts into solid Na_2_O suitable for subsequent vitrification. Sodium nitrate was explicitly chosen as the model nitrate species because it is one of the most abundant nitrate salts in the simulated HLLW, and its thermal decomposition behaviour is representative of the broader class of alkali and alkaline-earth nitrates present in the multicomponent waste mixture.

The use of sucrose as the organic reductant follows the established carbothermic denitration chemistry. Recent experimental work by Li et al. has confirmed that sucrose-based denitration effectively converts nitrates in simulated high-level liquid waste into solid oxides at calcination temperatures above 500 °C, with a sucrose-to-nitrate molar ratio of 1: 8 providing complete denitration [29]. The stoichiometry adopted in the present reactions is consistent with these experimental findings.

In the simulation, the solid product Na_2_O is treated as a discrete species phase within the gas-solid coupling framework. The reaction efficiency is evaluated based on its mass fraction distribution throughout the domain. The Arrhenius parameters for the two principal reactions are summarised in Table 2.

**Table 2:** Summary of global reactions and Arrhenius parameters used in the CFD model.

Reaction	*A* (s^-1^)	*Eₐ* (kJ mol^-1^)	*β*	ΔHᵣ (kJ mol^-1^)
48 HNO_3_ + C_12_H_22_O₁₁ → 48 NO_2_ + 12 CO_2_ + 35 H_2_O	1.0 × 10^10^	100	0	−1.2 × 10^3^
48 NaNO_3_ + C_12_H_22_O₁₁ → 24 Na_2_O + 48 NO_2_ + 12 CO_2_ + 11 H_2_O	2.0 × 10^12^	180	0	9.1 × 10^3^

The reaction rate for reaction i is computed as


$$\begin{array}{c} R_{i}=A_{i}T^{\beta_{i}}\text{exp}(-\frac{E_{a,i}}{RT})\prod_{j}C_{j}^{n_{ij}} \end{array}$$

where $C_{j}$ is the molar concentration of reactant j and $n_{ij}$ is the corresponding reaction order. Both reactions are assumed to be first-order with respect to each reactant (nitric acid or sodium nitrate, and sucrose), giving an overall second-order kinetics. The pre‑exponential factors and activation energies were estimated from typical values reported for nitrate decomposition and carbothermic reduction under high‑temperature calcination conditions and are valid over 473–1273 K. Since these kinetic constants have not been measured for the exact multicomponent waste composition investigated here, they introduce an inherent uncertainty into the quantitative reaction rates; a detailed discussion of the implications of this uncertainty is provided in Section 2.6. The enthalpy of reaction ΔHᵣ represents the net thermal effect of the coupled decomposition–oxidation process and is applied as a volumetric source term in the energy equation.

The thermophysical properties of all pure species required by the species-transport and energy equations are provided in Table 3.

**Table 3:** Thermophysical properties of pure species at 800 °C (1073 K) used in the simulations.

Species	M (g mol^-1^)	ρ (kg m^-3^)	c_p_ (J kg^-1^K^-1^)	k (W m^-1^ K^-1^)	D (m² s ^-1^)
HNO_3_	63.01	1512	1740	0.30	1.2 × 10^-5^
NaNO_3_	84.99	2260	1040	0.50	8.0 × 10^-6^
C_12_H_22_O₁₁	342.30	1580	1240	0.25	5.0 × 10^-6^
H_2_O (vap)	18.02	ideal-gas	2014	0.026	2.6 × 10^-5^
NO_2_	46.01	ideal-gas	840	0.020	2.0 × 10^-5^
CO_2_	44.01	ideal-gas	840	0.016	2.1 × 10^-5^
Na_2_O (s)	61.98	2270	960	0.80	1.0 × 10^-6^
N_2_	28.01	ideal-gas	1040	0.026	2.2 × 10^-5^
O_2_	32.00	ideal-gas	920	0.027	2.3 × 10^-5^

M = molecular weight; ρ = density (the ideal-gas law is used for gaseous species at the operating pressure; a constant density is assigned to solid Na_2_O); cₚ = specific heat at constant pressure; k = thermal conductivity; D = mass diffusivity in the gas mixture. Values are taken from standard thermodynamic tables and published data for nitrate–sucrose calcination systems.

In the present species‑transport model, Na_2_O is treated as a transported chemical species; although it is physically solid at the calcination temperature, solid‑particle nucleation, growth, and deposition on the reactor walls are not explicitly simulated. Consequently, the Na_2_O mass fraction reported throughout this work should be interpreted as an indicator of local chemical conversion rather than a direct measure of solid deposit. The formation and wall-bound interaction of solid particles will be the subject of future studies incorporating a discrete-phase or particle-tracking model.

### 2.4. Porous media model

The lateral off-gas filtration zone was modelled as an isotropic porous medium with a thickness of 10 mm. The flow resistance is implemented by adding a momentum sink term $S_{i}$ to the Navier–Stokes equations following the Darcy–Forchheimer law:


$$\begin{array}{c} S_{i}=-\left(\frac{\mu }{\alpha }v_{i}+C_{\text{2}}\frac{\text{1}}{\text{2}}\rho |v|v_{i}\right) \end{array}$$

where 1/α is the viscous resistance coefficient and $C_{\text{2}}$ is the inertial resistance coefficient. For the baseline porosity of ϕ=0.8, both coefficients were set to 400 m^-1^, corresponding to the sintered metal fibre filter used in the experimental apparatus. For the parametric sweep (ϕ=0 to 1.0), the coefficients at other porosities were derived from the Ergun equation, with its parameters calibrated to recover exactly 400 m^-1^ at the baseline porosity. The permeability αis obtained as the reciprocal of the viscous resistance. The resulting coefficients are summarised in Table 4.

**Table 4:** Porous-media parameters used in the simulations.

Porosity, φ	Viscous resistance, 1/α (m^-2^)	Inertial resistance, C_2_ (m^-1^)	Permeability, α (m²)
0.0 (blocked)	—	—	—
0.2	4.10 × 10^5^	1.19 × 10⁴	2.44 × 10^-6^
0.4	2.88 × 10⁴	1.12 × 10³	3.47 × 10^-5^
0.6	3.79 × 10³	2.21 × 10²	2.64 × 10 ^-4^
0.8 (baseline)	4.00 × 10²	4.00 × 10²	2.50 × 10^-3^
1.0 (fully open)	0	0	∞

The baseline coefficients at ϕ=0.8 were set to 400 m^-1^, matching the sintered metal fibre filter used in the experimental apparatus. The coefficients at other porosities were derived from the same Ergun-type relation after calibration to the baseline condition. For ϕ=0 the layer is impermeable; for ϕ=1 the porous resistance is removed.

### 2.5. Boundary conditions and parameter space definition

The top feed inlet was defined as a velocity inlet with a baseline flow rate of 8 L/h (velocity gradient: 0.001–0.2 m/s). The mixture comprised nitric acid (0.0801), sucrose (0.1203), nitrates (0.1184), and water (0.6812) by mass fraction. The calcination chamber (310S stainless steel) and collection hopper (304 stainless steel) were treated as constant temperature thermal boundaries, evaluated from 200 °C to 1200 °C (baseline: 800 °C). The lateral filtration zone porosity was varied from 0 to 1.0 (baseline: 0.8), and a 0 Pa gauge pressure outlet was applied. Computations were executed using a pressure-based steady-state solver with the SIMPLE algorithm. Second-order upwind discretization schemes were applied for all spatial variables, ensuring a stringent residual convergence criterion of 10^-4^.

### 2.6. Model validation

**Fig 4:**
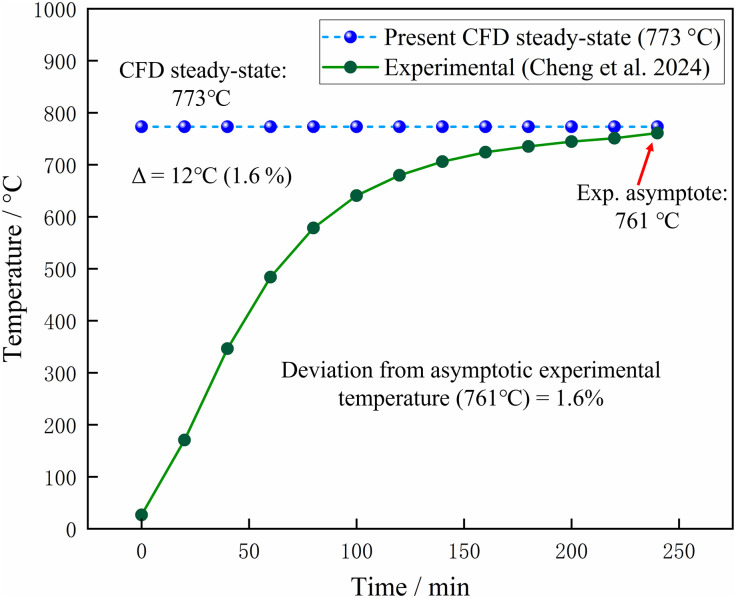
Validation of the simulated thermal field against experimental data.

To assess the reliability of the numerical framework, the simulated thermal field was compared with experimental data obtained on the same apparatus. Cheng et al. (2024) reported transient temperature measurements during the heating of the identical spray calciner [25]. Under the baseline wall‑temperature condition of 800 °C, their measurements show that the centre‑line temperature asymptotically approaches 761 °C after 240 min of heating. The present steady‑state CFD simulation predicts a centre‑line gas temperature of 773 °C, corresponding to a deviation of only 1.6%. The comparison is shown in Fig 4, where the experimental heating curve is plotted together with the steady‑state CFD result (dashed line). This close agreement confirms that the coupled heat‑transfer and fluid‑flow characteristics are accurately captured.

Model limitations and uncertainties. Although the thermal field has been successfully validated, several modelling assumptions introduce uncertainties that should be considered when interpreting the chemical conversion results. First, the Arrhenius parameters used for the two global reactions (Table 2) are literature‑based estimates rather than experimentally determined rates for the specific nitrate–sucrose waste solution. Variations in the pre‑exponential factors or activation energies could quantitatively affect the predicted Na_2_O mass fraction and shift the apparent temperature threshold at which NaNO_3_ decomposition becomes kinetically favourable. Second, the species‑transport model treats Na_2_O as a chemically inert transported scalar; solid‑particle nucleation, growth, and deposition on the reactor walls are not explicitly simulated. The reported Na_2_O mass fraction should therefore be interpreted as an indicator of local chemical conversion rather than a direct measure of solid accumulation. In addition, the model assumes ideal‑gas behaviour for all gaseous species and second‑order kinetics for the reactions. Finally, the steady‑state formulation, although appropriate for the continuously operated calciner under parametric comparison, inherently neglects transient phenomena such as start‑up heating, cyclic vibration‑induced solids detachment, and gradual fouling of the porous filter, which may be relevant for long‑term operational assessment. A formal quantitative uncertainty propagation (e.g., Monte‑Carlo sampling of kinetic parameters) and dedicated transient simulations are beyond the scope of this parametric study but represent important directions for future work.

## 3. Results and discussion

Utilizing the established three-dimensional numerical model of spray calcination, this chapter systematically investigates the effects of four critical process parameters—wall heating temperature, porous media porosity, inlet velocity, and waste liquid component concentration—on the generation characteristics of the reaction product, sodium oxide (Na_2_O). Through quantitative analysis of the simulation data, the coupling mechanisms between various physical field variables and chemical reaction rates are elucidated. It should be noted that the parameter-induced variations in Na_2_O mass fraction reported in the following sections are substantially larger than the quantified numerical uncertainty (mesh-related variation < 2%, as documented in Section 2.1; convergence-related variation < 0.5%, as specified in Section 2.2.1). The observed trends are therefore robust and not artefacts of numerical noise.

### 3.1. Mechanism of wall temperature on thermochemical conversion efficiency

Wall temperature fundamentally dictates the thermochemical kinetics within the reactor. Simulation results confirm a pronounced positive correlation between wall temperature and product yield. As the wall temperature is incrementally elevated from 200 °C to 1200 °C, the average mass fraction of Na_2_O at the monitoring point exhibits a non-linear increase from 0.089 to 0.360.

Kinetically, the endothermic nitrate decomposition strictly adheres to the Arrhenius law. In the low-temperature regime (200–600 °C), insufficient molecular kinetic energy fails to overcome the activation barrier, strictly constraining the reaction rate. Consequently, the chemical reaction rate is constrained, resulting in sluggish growth in product yield (increasing only from 0.089 to 0.169). However, once the temperature breaches 800 °C, the high-temperature environment markedly enhances the evaporation and heat transfer efficiency at the droplet surface. The reaction rate constant rises exponentially, enabling the rapid pyrolytic conversion of nitrates. The data reveals that the product mass fraction reaches 0.276 at 800 °C, narrowing the gap with the 0.304 achieved under the 1000 °C condition. This indicates that 800 °C represents the thermodynamic critical threshold (or inflection point) for this reaction system. At this temperature, an optimal calcination performance can be attained while concurrently circumventing the excessive energy consumption and severe material corrosion associated with excessively high temperatures.

**Fig 5:**
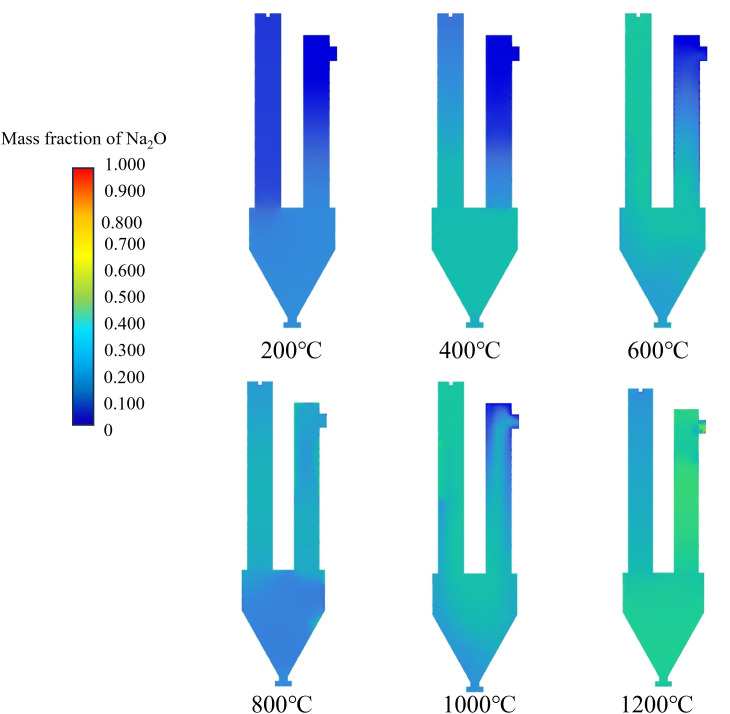
Contours of Na_2_O mass fraction distribution at various wall heating temperatures.

**Fig 6:**
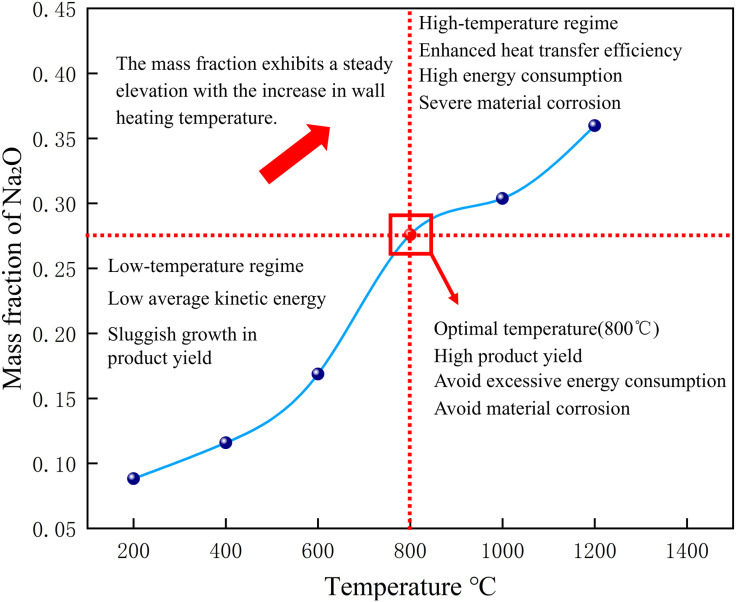
Variation of the average Na_2_O mass fraction as a function of wall heating temperature.

The spatial distribution of Na_2_O mass fraction at different wall heating temperatures is presented in Fig 5, and the corresponding variation of the average mass fraction is plotted in Fig 6.

### 3.2. Regulatory role of porous media porosity on flow field and product deposition

The porous media filtration zone serves as the frontal barrier for off-gas treatment, and its pore structure parameters exert a crucial secondary regulatory effect on the internal hydrodynamic characteristics of the apparatus. Numerical simulations reveal that as the porosity increases from 0 (completely blocked) to 1.0 (fully permeable), the generation of Na_2_O exhibits a fluctuating upward trend; however, within the broad flow channel range of 0.2 to 0.8, its growth slope tends to flatten.

This phenomenon elucidates the dual mechanism of porosity on “residence time” and “flow field perturbation”. Although a lower porosity (<0.2) increases the resistance of the gas flow through the filtration layer and significantly prolongs the physical residence time of reactants in the high-temperature zone, excessive backpressure leads to flow field turbulence, making it prone to form vortex dead zones near the walls, which could lead to non‑uniform product distribution and potential solids accumulation. Conversely, when the porosity is around 0.8, a stable pressure gradient is established within the apparatus, ensuring smooth fluid flow. This not only guarantees adequate mixing of reactants with high‑temperature gases but also reduces the likelihood of flow stagnation that could otherwise promote solids accumulation. Therefore, from the perspective of maintaining the long-term stable operation of the apparatus, a porosity of approximately 0.8 represents the optimal structural parameter to balance filtration efficiency and reaction yield.

**Fig 7:**
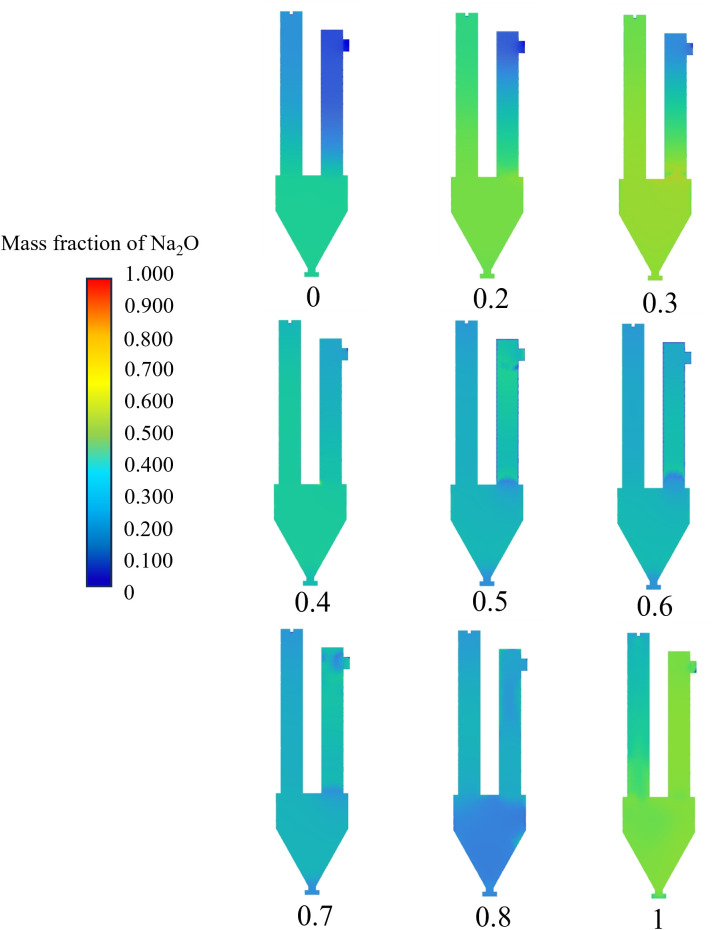
Contours of Na_2_O mass fraction distribution under varying porous media porosities.

**Fig 8:**
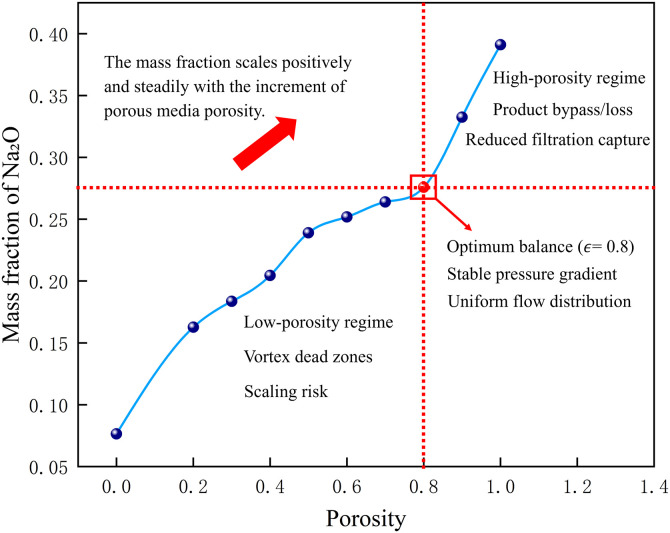
Variation of the average Na_2_O mass fraction as a function of porous media porosity.

These effects are illustrated in Fig 7 for the contours and Fig 8 for the average Na_2_O mass fraction versus porosity.

### 3.3. Influence of inlet velocity on transport characteristics and reaction limits

The inlet velocity dictates the initial momentum of the atomized waste liquid droplets and their spatial distribution within the reactor. Simulation results demonstrate that the variation of the Na_2_O mass fraction with inlet velocity exhibits a typical non-linear “rise-then-fall” characteristic, revealing a clear critical velocity threshold.

When the inlet velocity is at a relatively low level (0.001–0.01 m/s), an appropriate increase in velocity enhances the turbulent intensity of the jet and improves the gas-liquid two-phase mixing effect, thereby facilitating the chemical reaction. Within this regime, the Na_2_O mass fraction climbs steadily with increasing velocity, peaking at 0.287 at 0.01 m/s. However, as the velocity further increases to 0.05 m/s and up to 0.2 m/s, the product mass fraction drops precipitously to 0.229 and 0.170, respectively. This is attributed to the fact that excessively high velocities drastically curtail the actual residence time of reactants in the high-temperature reaction zone—a phenomenon termed the “kinetic breakthrough” effect, wherein reactants are carried away from the core reaction zone by the carrier gas before absorbing sufficient heat to complete the decomposition reaction. This result quantitatively dictates that the velocity corresponding to the optimal processing throughput for the current geometry of the apparatus should be strictly controlled at around 0.01 m/s.

To support this interpretation quantitatively, the nominal residence time τ of the fluid in the high-temperature zone (the region above the porous filter, with an axial length ≈ 0.4 m) was estimated as τ = L/ v_in_. For the inlet velocities investigated, this yields τ ≈ 40 s at 0.01 m s^-1^, 8 s at 0.05 m s^-1^, 4 s at 0.10 m s^-1^, and 2 s at 0.20 m s^-1^. The 20-fold monotonic decrease in nominal residence time directly explains the sharp decline in Na_2_O mass fraction at higher velocities. Although the actual residence time distribution is complicated by recirculation and backmixing, these estimated values support the conclusion that sufficient exposure to high temperature is essential for complete calcination.

**Fig 9:**
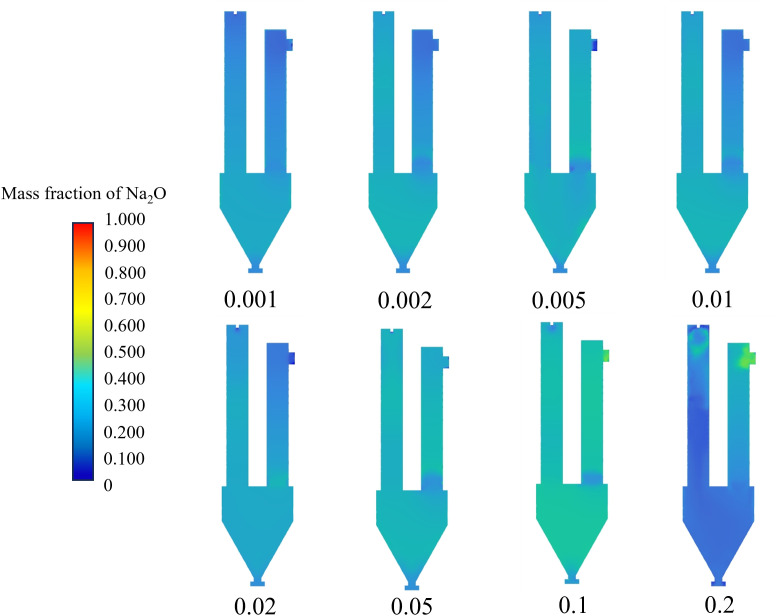
Contours of Na_2_O mass fraction distribution at different inlet velocities.

**Fig 10:**
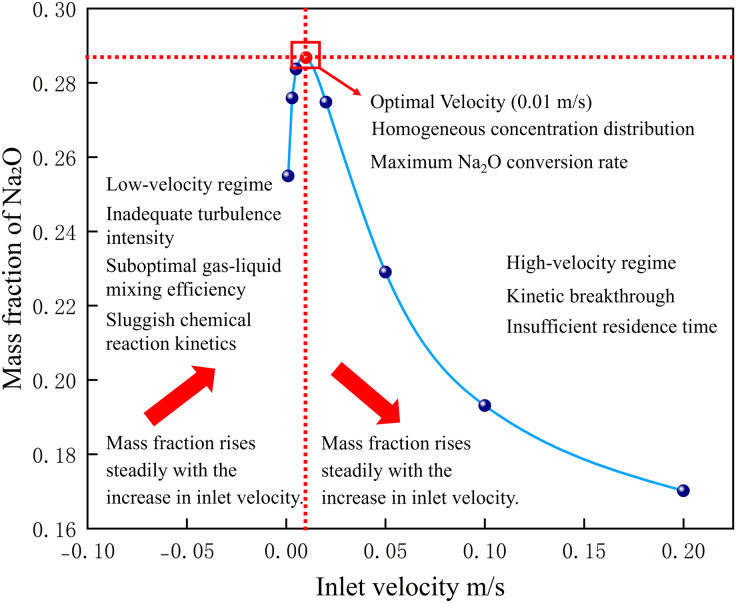
Variation of the average Na_2_O mass fraction as a function of inlet velocity.

The visualised flow structures and the velocity-dependent Na_2_O yield are shown in Fig 9 and Fig 10, respectively.

### 3.4. Sensitivity analysis of waste liquid component concentration on reaction kinetics

In the following analysis, “diluted” refers to halving the mass fraction of the reactant of interest (e.g., sucrose) while proportionally increasing the water content to maintain the same total feed rate; “concentrated” refers to doubling the reactant mass fraction under the same total feed constraint. The percentage enhancement is defined as $\text{Enhancement}\left(\%\right)=\frac{Y_{\text{conc}}-Y_{\text{dil}}}{Y_{\text{dil}}}\times \text{1}\text{0}\text{0}\%$, where Y is the area-averaged Na_2_O mass fraction at the reactor outlet.

A concentration sensitivity analysis of the three primary components—nitric acid, sucrose, and nitrates—in the simulated high-level liquid waste indicates that variations in different component concentrations contribute to final product generation with significant disparities, exhibiting strong stoichiometric dependency. The detailed parameters are listed in Table 5.

**Table 5:** Mean mass fraction of Na_2_O produced under various reactant concentration.

Reactant concentration	Mean Na_2_O mass fraction (Concentrated)	Mean Na_2_O mass fraction (Diluted)
Nitric acid	0.289	0.251
Sucrose	0.304	0.248
Sodium nitrate	0.286	0.257

Simulation data reveal that under the same concentration multiple (1-fold concentration), the elevation of sucrose concentration exerts the most drastic promoting effect on Na_2_O generation. Specifically, the enhancement amplitude of the product mass fraction after sucrose concentration is approximately 115% higher than that of nitric acid and 180% higher than that of nitrates. This conclusion profoundly reflects the essence of this reaction system: the carbothermic reduction reaction, utilizing sucrose as the reducing agent, is the rate-determining step of the overall conversion. Sucrose not only provides a reducing atmosphere, but the heat and gases generated by its decomposition also assist in refining the droplets. Conversely, when the components are diluted, the decline in yield caused by the reduction in sucrose concentration is also the most significant. Therefore, in actual process formulation adjustments, prioritizing the optimization of the reductant (sucrose) addition ratio is the most effective approach to elevate the yield of the solidified product.

It should be noted that the present two-level comparison (diluted vs. concentrated) captures the first-order sensitivity of Na_2_O yield to each reactant concentration. A more finely resolved concentration sweep with multiple intermediate levels is recommended for future work to fully characterise the concentration–response relationships and to identify any potential nonlinearities within the intermediate concentration range.

**Fig 11:**
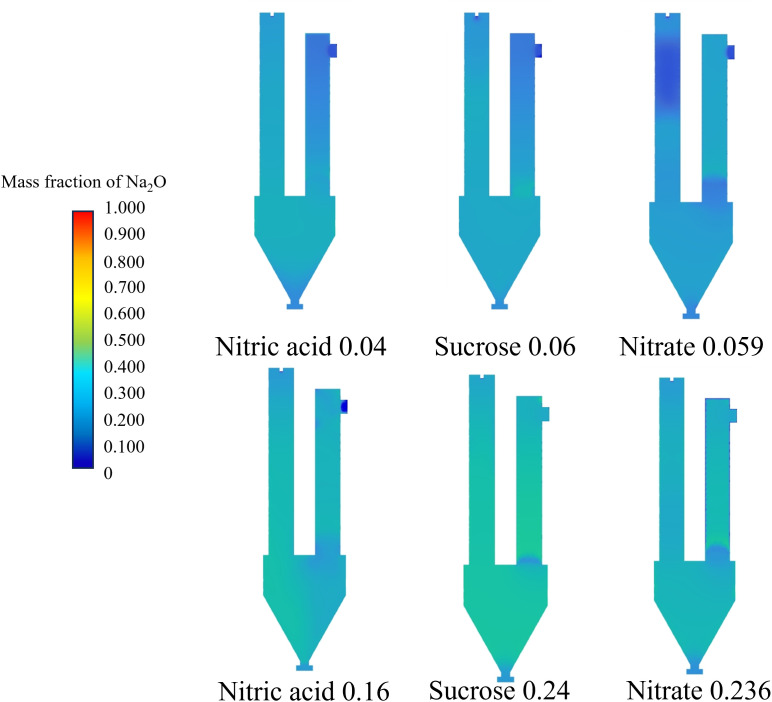
Contours of Na_2_O mass fraction distribution under varying concentrations of nitric acid, sucrose, and nitrates.

**Fig 12:**
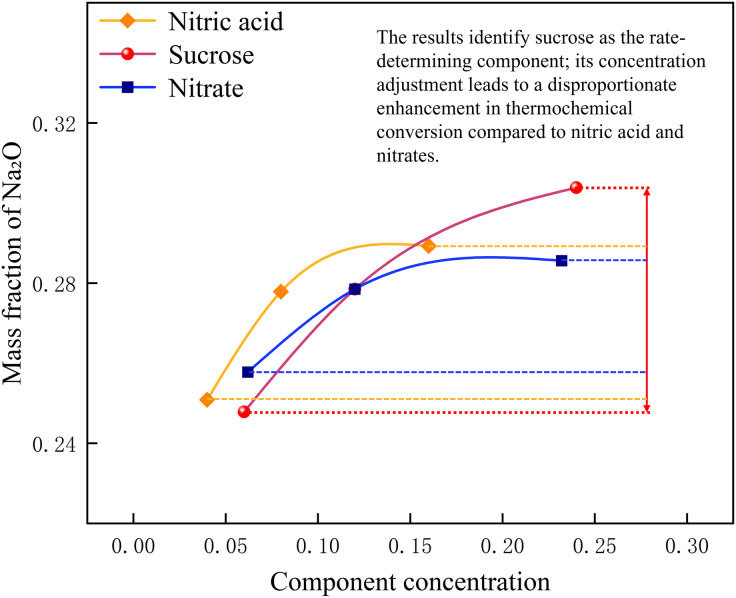
Variation of the average Na_2_O mass fraction in response to adjusting reactant component concentrations.

The corresponding spatial distributions are given in Fig 11, and the quantitative comparison is summarised in Fig 12.

### 3.5. Interaction effects among operating parameters

Although the preceding analysis examined each parameter independently, the four factors—wall temperature, porous media porosity, inlet velocity, and reactant concentration—are intrinsically coupled through the underlying thermal, flow, and mass-transfer mechanisms. The nominal residence time, which is jointly determined by the inlet velocity and the porous-media resistance, serves as a central coupling variable. Sufficient residence time is a prerequisite for the reactants to absorb heat and reach the activation temperature, and only when this thermal-kinetic condition is satisfied does the reactant concentration become the rate-controlling factor. The porosity-induced flow restructuring further modulates the spatial uniformity of the temperature field: recirculation zones created at low porosity produce local cold spots that suppress reaction locally, while a uniform flow field at ≈ 0.8 porosity ensures efficient utilisation of the high-temperature environment. Additionally, the two global reactions compete for the same reductant (sucrose), creating a stoichiometric coupling between the nitric acid and sodium nitrate decomposition pathways. These interaction mechanisms indicate that a formal multi-parameter optimisation would be a valuable extension of the current work.

Based on the maximum Na_2_O yield variation induced over each parameter’s investigated range, the qualitative sensitivity ranking is: wall temperature (dominant, factor > 4)> inlet velocity (∼ 70% variation, with a narrow favourable window)> sucrose concentration (∼ 22% enhancement upon doubling, the most influential concentration parameter)> porosity (significant mainly at the extremes of the 0–1 range, but with a relatively flat plateau within 0.2–0.8).

## 4. Conclusions

This research developed a high-fidelity three-dimensional numerical simulation platform under multi-physics coupling, targeting the spray calcination process in the pretreatment of HLLW vitrification. By integrating the species transport model with Arrhenius chemical kinetic parameters, this work systematically elucidated the thermochemical conversion mechanisms and spatial distribution characteristics of the complex “nitric acid-sucrose-nitrate” system within the high-temperature reaction zone. Compared to conventional studies, this paper quantitatively delineated the non-linear mapping relationship between external physical fields and internal chemical phase transitions through multidimensional parameter sensitivity analysis. The research outcomes clarify the rate‑determining steps and thermodynamic limiting factors during the calcination process and provide simulation‑based guidance for process parameter selection. The main conclusions are summarized as follows:

1. Thermodynamic Driving Mechanism and Temperature Response Characteristics: The research confirms that wall heating temperature is the key factor regulating the kinetic reaction rate. As the boundary temperature increases linearly from 200 °C to 1200 °C, the generation of the core product Na_2_O exhibits a distinct stepwise growth, with the average mass fraction leaping from 0.089 to 0.360. For the present reactor geometry and within the investigated range of 200–1200 °C, 800 °C represents the thermodynamic threshold above which the energy input is sufficient to overcome the activation barrier, enabling stable thermal conversion.
2. Regulatory Role of Porous Media Topology on Flow Field State: Through a parametric sweep of the porosity (ϵ) in the off-gas filtration zone, a significant secondary regulatory effect on the reaction yield was discovered. Within the broad range of ϵ from 0.2 to 0.8, the pressure gradient and species concentration gradient within the apparatus exhibit excellent decoupling characteristics, ensuring process robustness. Under the geometric and operating conditions examined, a porosity of ϵ = 0.8 yields the most favourable balance between flow resistance and reaction residence time, contributing to uniform internal flow fields and a reduced risk of localized solids accumulation.
3. Quantitative Relationship Between Residence Time Distribution and Velocity Limits: The inlet velocity dictates the completeness of thermochemical conversion by altering the residence time of reactants in the high-temperature zone. Within the parameter range studied, the Na_2_O mass fraction first increases with inlet velocity, reaching a maximum of 0.287 at approximately 0.01 m s^-1^, and then decreases sharply when the velocity exceeds 0.05 m s^-1^. This behaviour suggests that, for the present configuration, a velocity around 0.01 m s^-1^ is favourable, while velocities above 0.05 m s^-1^ may lead to insufficient residence time and reduced conversion.
4. Component Kinetic Sensitivity and Formulation Optimization Criteria: Sensitivity analysis based on mass fraction variations indicates that the reaction system exhibits a pronounced “reductant-limited” characteristic. Under the same concentration gradient, the contribution weight of sucrose concentration to the final equilibrium product is the most prominent, with its enhancement amplitude on product mass fraction being 115% and 180% higher than that of nitric acid and nitrates, respectively. These results indicate that, among the parameters examined, the sucrose proportion has the strongest influence on Na_2_O yield, suggesting that adjusting the reductant ratio is an effective way to improve conversion.

The four parameters investigated do not act independently; the coupling among wall temperature, residence time, and flow uniformity determines the operating window for effective calcination within the parameter ranges studied. The classification into a thermally limited regime (low temperature) and a transport-limited regime (high velocity) provides a unified framework for interpreting these coupled effects.

## Supporting information

S1 File Numerical data underlying Figs 6, 8, and 10. (XLSX)
